# Carbon price volatility: The case of China

**DOI:** 10.1371/journal.pone.0205317

**Published:** 2018-10-10

**Authors:** Yinpeng Zhang, Zhixin Liu, Yingying Xu

**Affiliations:** 1 School of Economics and Management, Beihang University, Beijing, China; 2 Donlinks School of Economics and Management, University of Science & Technology Beijing, Beijing, China; Vilnius University, LITHUANIA

## Abstract

Based on carbon spot prices selected from seven carbon pilots, we assess the financial performances related to carbon volatility in China on the overall perspective. According to the results, the Chinese carbon market fluctuated severely at the beginning of carbon trading, but has stabilised in general, despite several dramatic changes related to ‘yearly compliance events’. Long-term memory exists in the volatility series. Moreover, asymmetry exists in the Chinese carbon market, and volatility reacts more severely to good news than to bad news. Finally, we discuss our empirical results, and make certain suggestions regarding firms’ awareness, international cooperation and individual investors not only for policy makers in China but also for other developing countries who are contemplating either commencing carbon trading or improving the current market.

## Introduction

With the onset of industrialisation, greenhouse gas emissions increased rapidly, and they are now of great concern globally because of their potential impacts on climate change and human sustainable development. According to the United Nations Framework Convention on Climate Change (UNFCCC) and the Kyoto Protocol, an Emission Trading Scheme (ETS) is regarded as the most cost-effective way to reduce emissions. Developing countries, including China, do not have to undertake mandatory emission reduction tasks. China, as the largest developing country and the largest emitter of greenhouse gases, has established seven carbon emission pilots in her mainland and expects to establish a unified national carbon market to ease the pressures from international climate negotiations. After the announcement made by the National Development and Reform Commission (NDRC) on 19 December 2017, the establishment of a national ETS is approaching. Thus, it is of great priority to assess the performances of the seven pilots on the overall perspective for the forthcoming national ETS. In this paper, we focus on assessing the volatility in the carbon market, along with previous studies [1–2], but from an overall perspective, rather than focusing on an individual market.

The ETS is a government-established and market-mechanism-based strategy for controlling carbon emissions. Commonly, the government sets the amount of permitted carbon dioxide and allocates the quotas to certain firms. Emitters constrained by ETS need to hold allowances that are greater than the amount of carbon dioxide they produce. Emitters who can reduce their carbon emissions can sell excess allowances to those entities with insufficient allowances. [3]

Ever since the first and the largest carbon market, i.e., European Union Emission Trading Scheme (EU-ETS) was established, many studies have assessed its performance [4–6]. Nevertheless, concerns and discussions have been raised within developing countries, e.g., China [7–10], about carbon market. However, to the best of our knowledge, the volatility of the Chinese carbon market from an overall perspective remains understudied empirically even though the national ETS is going to be established. Carbon products traded in different pilots are essentially the same product. Therefore, it is reasonable to integrate all the seven carbon pilots to represent the overall Chinese carbon market. In this paper, we integrate carbon spot prices selected from the seven carbon pilots to fill the gap by investigating the carbon price volatility through the exponential generalized autoregressive conditional heteroscedastic (E-GARCH) model, the Augmented Dickey Fuller (ADF)-Kwiatkowski Phillips Schmidt Shin (KPSS) joint test, and rescaled range (R/S) analysis.

This paper makes the following contributions. First, the previous literature has generally focused on a single pilot, i.e., Beijing, Tianjin, Hubei, Shanghai, Shenzhen, Chongqing, or Guangdong. Conclusions and suggestions are proposed based on the details of a single market. It is questionable whether these results apply to the overall carbon market. According to the NDRC, equal weight will be assigned to each pilot [11]; thus, this paper calculates the average price of the seven carbon pilots to represent a national carbon price for investigating the overall Chinese carbon market. Such integration may overcome potential deficiencies induced by lower transaction volumes in certain pilots. Second, based on the integration of the pilots, the volatility series and the characteristics, i.e., the long-term memory and asymmetry are investigated systematically through the E-GARCH model, the autocorrelation coefficient, the ADF-KPSS joint test, and the Hurst index. We discuss the reasons for the volatility series and its characteristics, which benefit policy makers both in China and other developing countries in terms of carbon trading.

In this paper, we collect the closing prices of spot carbon from the first trading day, 28 November 2013, of the seven carbon pilots to22 May 2017. Our findings suggest that the Chinese carbon market fluctuated severely at the beginning of carbon trading, but has stabilised, despite some dramatic changes related to ‘yearly compliance events’. Additionally, long-term memory exists in volatility series. Asymmetry exists in the carbon market, and volatility reacts more strongly to good news than to bad news. The reminder of this paper is organized as follows, Section 2 is a literature review. Section 3 introduces some basic situations of the Chinese carbon market. Section 4 presents the methodology. Section 5 shows the empirical results and Section 6 discusses the results, and Section 7 concludes this paper.

## Literature review

Among all the existing literature connected with ETSs, the EU-ETS is the focus. An important line of the research on EU-ETS refers to the performance, i.e., volatility, of the EU-ETS carbon market. Ellerman [12], Mansanet-Bataller [13] and Alberola [14] argued that the regulatory announcements and over-allocation of allowances result in volatility jumps. Benz [15] points out that the return series of the carbon spot price may be ‘fat-tailed’ and models the volatility with skewness and kurtosis. Chevallier [5] reports on the non-linear correlations inside the volatility series in the EU-ETS. Sanin [6] introduces an additive stochastic jump process in the modelling of the volatility of the carbon price. Chevallier [16] points out that the introduction of a carbon option modifies the volatility of carbon in the EU. All the literature aims to assess the performances of the EU-ETS carbon market through volatility, thus contributing to the understanding of this relatively immature and emerging market.

Another line of research refers to the correlations between the EU-ETS carbon markets and other financial markets. Luo [17] and Zhang [18] investigate the dynamic correlations between the EU-ETS carbon market, the commodity markets, and the stock markets for the second and third phase of the EU-ETS respectively. Yu [19] focuses on the Granger causality between the carbon future market and the crude oil market in the short, medium, and long term. Oestreich [20] argues that firms that received free quotas outperformed those that did not and that the EU-ETS generates a ‘carbon premium’, which can be accounted by a higher cash flows due to the free quotas. In-depth study, Moreno [21] points out that such impacts are sector-specific. Bushnell [22] investigates how the EU-ETS affects profits and argues that the increase of polluting prices will be reflected in the product prices, resulting in the revenue increasing. These studies focus on the connections between the EU-ETS carbon markets and other financial markets, contributing to the understanding of the emerging financial market and portfolio constructions.

In addition to the studies looking at the EU-ETS, a few studies have focused on the carbon markets in developing countries, e.g., China. Concerns and discussions have been raised within China about the current carbon pilot markets [7–10]. An important part of these concerns is to assess the current performance of the carbon markets. Yang [23] points out that the domestic carbon market is an authority-manipulated system. Cong [1] assesses the financial performances of carbon markets by focusing on the volatility series in the Shenzhen pilot carbon market in China. He finds that volatility is negatively connected with return, which should never appear in an efficient market. However, Zhao [24] holds a different view. He sampled the Beijing, Tianjin, Shanghai, and Shenzhen carbon pilots and suggests weak form efficiency of the carbon markets. In addition, Zhao [24] find evidence of the connection between the increased trading volume and the improvement of market efficiency. Based on the carbon spot prices selected from the seven carbon pilots, Chang [2] argues that there are leverage effects in Chinese carbon market. Fan [11] investigates the linkages between four carbon pilots and macro factors and argues that the fundamental of carbon price is weak and immature. These studies of the Chinese carbon market focus on one single carbon pilot and provide little evidence regarding the overall market even the nation ETS is going to be established, the existing results based on one single pilot may be biased.

So, in this paper, we fill the gap by integrating the closing prices of all seven carbon pilot markets in China. Specifically, we focus on the volatility. To the best of our knowledge, this is the first study focusing on the overall volatility of the Chinese carbon market.

## Carbon trading in China

Seven pilots, aimed at accumulating experience for the unified mandatory carbon market in China, have been established under the supervision of NDRC. They are Shenzhen, Beijing, Shanghai, Tianjin, Guangdong, Hubei, and Chongqing respectively. On 19 June 2013, the Shenzhen Emission Exchange ushered in its first market day, which saw carbon trading go from theory to practice in China.

Currently, all the seven carbon pilots are established based on the experience of the EU-ETS. The constrained firms have been identified by the government. Similarly, quotas have been identified by the government based on the knowledge of the target firms. Besides, are the quotas are allocated for free or through auctions. The government has organized a professional third party to verify historical data on carbon emissions from target companies. There are ‘yearly compliance events’ to check the firms for their allowances. Meanwhile, all the pilots have adopted the ‘cap-and-trade’. The seven carbon pilots constrain more than 2,000 companies, including typical heavy industries, i.e., steel, power, petrochemical, cement, and other non-industrial sectors such as construction, transportation, and services.

Since the establishment of the first carbon pilot, carbon pilots have operated for several years and have achieved remarkable success. Nowadays, the Hubei carbon pilot market has become the largest carbon market in Asia; besides, the trading volume and the turnover have increased rapidly [25]. Approximately 1,200 million tons of quotas are allocated to the seven carbon pilots each year. Once a unified national carbon market is established, it will become the largest carbon market in the world.

## Methodology

In this section, we introduce certain basic concepts that are used to assess the volatility.

### 4.1 Exponential GARCH (E-GARCH) model

The variance is widely used to measure volatility. However, the most successful way to model volatility for financial time series is the GARCH model [26]. The standard GARCH model can be written as follows.

$$\mathrm{Mean}\;\mathrm{equation}:\;y_{t}=c_{1}+\mu_{t}$$ (1)

$$\mathrm{Variance}\;\mathrm{equation}:\;\sigma_{t}^{2}=c_{2}+c_{3}\mu_{t-1}^{2}+c_{4}\sigma_{t-1}^{2}$$ (2)

where {*y*_*t*_} represents the target series, *c*_1_ is the conditional mean, *μ*_*t*_ is the residual, $\sigma_{t}^{2}$ is the conditional variance, *c*_2_ is a constant, and *c*_3_ and *c*_4_ are parameters that satisfy the conditions of *c*_3_ ≥ 0 and *c*_4_ ≥ 0. Despite the well describing for heteroscedasticity of the residual term, this model fails to reflect the impacts, i.e., information shock or leverage effects, of information shocks. Information asymmetry means the market’s different responses to good/bad news. Good or bad news can affect the volatility of the financial markets. If such impacts have no significant difference, the volatility can be regarded as symmetrical; otherwise, the volatility can be regarded as asymmetrical. To overcome the deficiency of the GARCH, the exponential GARCH (E-GARCH) model is proposed by Nelson [27]. As Chang [8] argues the leverage effects in the Chinese carbon market, thus, the E-GARCH model is chosen for modelling. The mean equation of E-GARCH is the same as in the GARCH model, but the variance equation has been updated. The variance equation can be briefly summarized as follows.

$$\ln\left(\sigma_{t}^{2}\right)=\omega +\beta \;\ln\left(\sigma_{t-1}^{2}\right)+\gamma *\mu_{t-1}/\sqrt{\sigma_{t-1}^{2}}+\alpha \left[\left|\mu_{t-1}\right|/\sqrt{\sigma_{t-1}^{2}}-\sqrt{2/\pi }\right]$$ (3)

Since $\ln\left(\sigma_{t}^{2}\right)$ can be negative, the E-GARCH model has no constraints on parameters, and ω, β, γ and *α* need not to be non-negative.

### 4.2 Serial correlation test

For a random time series {*x*_*t*_}, the coefficient between {*x*_*t*_} and {*x*_*t*−*l*_} is called the autocorrelation coefficient, in which *l* represents the time-lag. The autocorrelation coefficient is defined as:

$$r_{l}=cov(x_{t},x_{t-l})/(\sqrt{Var(x_{t})}\sqrt{Var(x_{t-l})})$$ (4)

where *cov* represents the operator of co-variance and *Var* is the operator for variance. The autocorrelation coefficient is used to describe the degree of correlation of the time series. In other words, the autocorrelation coefficient is used for measuring the impacts of historical data on current data. For a certain series, *r*_*l*_ cannot be calculated directly as we cannot achieve all the information of a time series. Specifically, we cannot determine the unknown parameters of *cov*(*x*_*t*_,*x*_*t*−*l*_) and *Var*(*x*_*t*_). In practice, we can only calculate the approximate value from a sample autoregressive coefficient to represent the autocorrelation coefficient by the following equation.

$$r_{k}=\sum_{t=1}^{n-k}\left(x_{t}-x)(x_{t+k}-x\right)/\sum_{t=1}^{n}{\left(x_{t}-x\right)}^{2}$$ (5)

where *r*_*k*_ represents the correlation coefficient, *x*_*t*_ represents the target sample series, and $x=(\sum_{t=1}^{n}x_{t})/n$ corresponds to the average value of the series.

If there is no memory in the target series, the correlation coefficient between the target series and the lagged series should be relatively small. If the magnitude of the coefficient is greater than zero, then the sequence may have short/long term memory. Quantitative testing is needed to identify the kind of memory.

### 4.3 Augmented Dickey Fuller (ADF)-Kwiatkowski Phillips Schmidt Shin (KPSS) joint test

The ADF unit root test is generalized from the DF test [28–30], and the KPSS test was introduced by Kwiatkowski [31]. Lee [32] extended the method to distinguish short-term memory and long-term memory of a time series. Both methods can test whether the target series is stationary, but the null hypothesis for the two methods is quite different. While the null hypothesis of the ADF test supposes that the target is not stationary and a unit root exists, the KPSS assumes the series to be stationary. Hence, the formations of the two tests are different from each other; these, can be summarized, respectively, as follows:

$$\mathrm{ADF}:\;△x_{t}=\gamma x_{t-1}+\alpha +\delta t+\sum_{i=1}^{p}\beta_{i}△x_{t-i}+\mu_{i}$$ (6)

$$\mathrm{KPSS}:\;x_{t}=\alpha_{t}+\beta t+\mu_{t}$$ (7)

In the above models, *x*_*t*_ represents the target series, *γ*, *α*, *δ*, *β*_*i*_, *α*_*t*_, and *β* are parameters, *p* refers to the lag length based on the statistical criterion, and *μ*_*i*_ and *μ*_*t*_ are the residuals. Given the different assumptions of the two models, the ADF-KPSS Joint Test can test the stationary state and long-term memory simultaneously. If {*x*_*t*_} rejects the ADF and accepts the KPSS, *x*_*t*_ is stationary. If {*x*_*t*_} rejects the ADF and the KPSS simultaneously, the series may have long-term memory.

### 4.4 R/S analysis

Long-term memory refers to a significant correlation between the target series and its lag, even when the lag length reaches a huge magnitude. Long-term memory in volatility series usually exists in a financial market, there usually exists long-term memory in volatility series [33–34]. The R/S analysis was developed by Hurst [35]. Mandelbrot [36] extended the method to finance and has made it the most commonly used method in testing for the memory state of a selected financial series. As there are few additional requirements on the research object, the Hurst index shows remarkable robustness. The classical R/S method can be summarized as follows.

Suppose that {*x*_*t*_} represents the target financial time series consisting *n* components, the Hurst Index *H*_*n*_ can be calculated as: ${\left(n/2\right)}^{H_{n}}=R_{n}/S_{n}$, where *R*_*n*_ and *S*_*n*_ are calculated as follows:

$$R_{n}=\max_{1\leq k\leq n}\sum_{j=1}^{k}\left(x_{j}-a\right)-\min_{1\leq k\leq n}\sum_{j=1}^{k}\left(x_{j}-a\right)$$ (8)

$$S_{n}=\sqrt{\sum_{j=1}^{n}{\left(x_{j}-a\right)}^{2}/n}$$ (9)

where *a* represents the average value of the series. If the Hurst index calculated from the R/S analysis locates in (0, 0.5), a short-term memory exists. If it locates in (0.5, 1), a long-term memory exists. If the Hurst index is equal to 0.5, the series follows a random walk [37].

## Empirical results

### 5.1 Data

**Fig 1:**
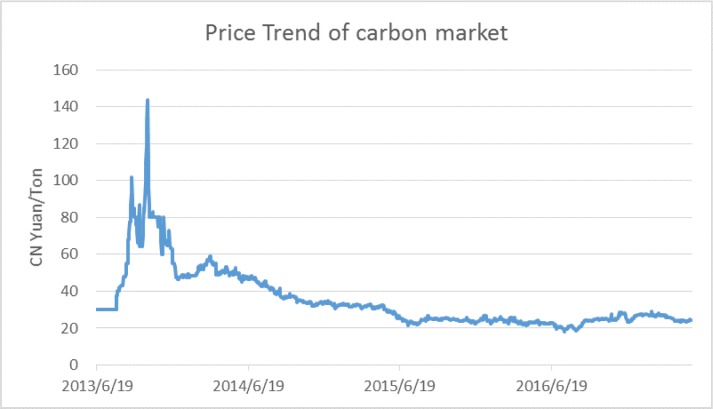
Price trend of the carbon market in China.

In this section, we collect the data of closing prices from the seven pilot carbon markets in China from the first trading day to 22 May 2017. The first trading day of the Shenzhen carbon pilot was 19 June 2013, and there are four types of emission allowances: SZA 2013, SZA 2014, SZA 2015, and SZA 2016. Given that each type may not be representative because of its potential low transaction volume, we calculate the average closing prices of the four to represent the Shenzhen carbon pilot. For the other six pilots, there is only one type of allowance traded in each market. For the Beijing carbon pilot, we collect the closing prices from the first trading day, 28 November 2013 to 22 May 2017. For Shanghai and Guangdong, we collect the closing prices from 19 December 2013 to 22 May 2017. For Tianjin, we collect the closing prices from 26 December 2013 to 22 May 2017. For Hubei, we collect closing prices from 2 April 2014 to 22 May 2017. For Chongqing, we collect closing prices from 19 June 2014 to 22 May 2017. We calculate the average closing prices of the seven pilots to represent the national carbon closing price, coving 1,191 trading days with all missing data deleted. The average closing price is calculated based on existing carbon pilots. Specifically, if one pilot is not established when calculating the average closing prices, it is not included in the calculation. We show the price trend in Fig 1.

As we can see from Fig 1, at the beginning of carbon trading, the closing price remains stable, after which the price increases to a high of more than 100 Yuan per ton. Later, the price begins to decrease sharply, but it is still higher than 60 Yuan per ton. After the decrease, the prices rise quickly to more than 140 Yuan per ton. Following this, the overall price trend declines. The price experiences a small increase in the first half of 2014. From the second half of 2014, the price is comparatively stable in the range of 20 to 30 Yuan per ton.

**Fig 2:**
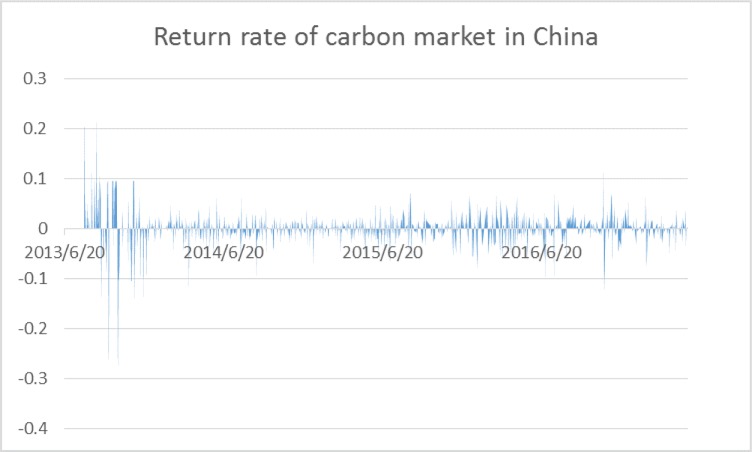
Return rate of the carbon market.

To reflect the price changes, we calculate the return rate of the carbon market using the equation of *r*_*t*_ = ln(*p*_*t*_/*p*_*t*−1_) and summarize the statistical properties of the return series in Table 1 and Fig 2.

**Table 1:** Descriptive statistics of the return series.

	Mean	Variance	Skewness	Kurtosis	Median	Min	Max
Value	-0.0002	0.0010	-1.1265	23.2826	0	-0.3059	0.2364

As we can see from Table 1 and Fig 2, the average return of carbon is not positive in China with a magnitude of -0.02%. Most of the daily returns are comparatively stable, as the minimum value reaches a low of -30.59% and the maximum value reaches a high of 23.64%. However, the variance is relatively small, with a magnitude of 0.0010. Besides, like most financial markets, the return series is not normally distributed, as the kurtosis exceeds three and reaches a magnitude of 23.2826. These data reveal the fat-tailed distribution of return series.

We cannot model the target series using the GARCH-family if it is not stationary. In this part, we test the stationary status of the return series. The results of the ADF-KPSS joint test are shown as follows.

As shown in Table 2, the results reject the null hypothesis of the ADF test, meaning that the target series is stationary. The absolute value of the ADF statistic is far greater than the absolute value of critical values. Besides, the results cannot reject the null hypothesis of the KPSS test, which regards the return series to be stationary. We confirm that the return series is stationary and can be used for the E-GARCH modelling.

**Table 2:** Results of return series for the ADP-KPSS joint test.

	Significance Level	ADF test	KPSS test
		-34.0109	0.1370
Critical values	1% level	-3.4356	0.7390
	5% level	-2.8638	0.4630
	10% level	-2.5680	0.3470

### 5.2 Results of EGARCH model

**Fig 3:**
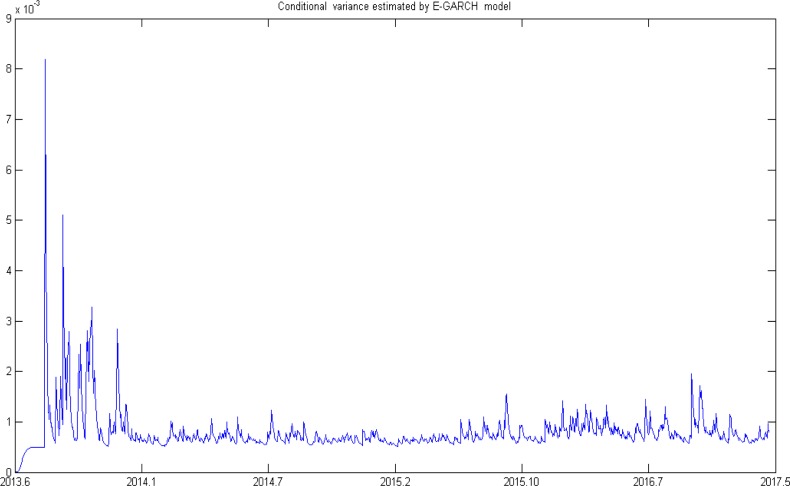
Conditional variance estimated by the E-GARCH model.

Fig 3 and Table 3 show the conditional variance based on the E-GARCH model.

**Table 3:** Descriptive statistics of the conditional variance series.

	Mean	Variance	Skewness	Kurtosis	Median	Min	Max
Value	0.0008	1.9*10–7	6.9274	83.3349	0.0007	0	0.0082

As shown in Fig 3, the conditional variance changes severely at the beginning of carbon trading, with the magnitude reaching a high of more than 8*10^−3^. From the first half of 2014, the volatility is stable. The conditional variance is below 1*10^−3^ most of the time. However, there is dramatic fluctuation in the second half of each year, reaching a high of 2*10^−3^. As shown in Table 3, the volatility series is relatively stable, as the mean of the series equals to 0.0008, the variance of the series is 1.9*10^−7^, the median is 0.0007, and the skewness equals to 6.9274.

### 5.3 Results of memory test

In this section, three different methods are adopted to test the memory state of the carbon volatility series in China.

**Fig 4:**
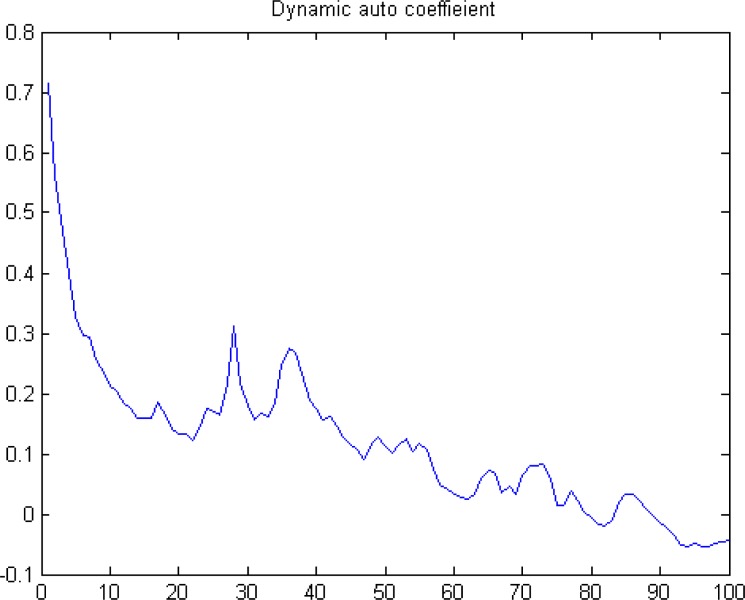
Auto-coefficients for different lags.

First, the serial correlation test. This method is simple and straightforward but subjective, as the critical result relies solely on the autoregressive coefficient. If memory exists, the auto-coefficient between the series and the lagged series should be positive for a certain lag length. If not, the series can be regarded as a random walk. We summarize the auto-coefficients of volatility series for different lags in Fig 4. As shown in Fig 4, the trend for the autoregressive-coefficient is declining, and the coefficient is positive even at the lag length of 80. Thus, long-term memory seems to exist.

Second, the ADF-KPSS joint test. If long-term memory exists in the volatility series, the results of the joint test should reject the ADF test and the KPSS test simultaneously. We show the results of the ADF-KPSS joint test as follows in Table 4.

**Table 4:** Results of volatility series for the ADP-KPSS joint test.

	Significance Level	ADF test	KPSS test
		-11.7903	0.4133
Critical values	1% level	-3.4356	0.7390
	5% level	-2.8638	0.4630
	10% level	-2.5680	0.3470

The result of the ADF unit root test shows that we should reject the null hypothesis and accept the alternative one as the absolute value of the ADF test statistic is 11.7903, which is greater than the critical values at either the 1%, 5%, or 10% significance levels. However, the KPSS test rejects the null hypothesis at the 10% significance level and accepts the alternative as the KPSS test statistic is 0.4133, which is greater than the critical value at the 10% significance level. The controversial results from the ADF-KPSS joint test show that long-term memory may exist in the volatility series of the Chinese carbon market.

Third, R/S analysis. If the Hurst index for R/S analysis locates in (0.5, 1), this refers to a long-term memory. The Hurst index for the volatility series, which is equal to 0.8661, exceeds 0.5. According to the literature, we confirm that a long-term memory, rather than a short-term memory, exists in the volatility series.

### 5.4 Asymmetry of volatility series

Campbell [38] and Schwert [39] point out that in a financial market, negative news usually causes greater shocks to volatility than good news. We compare such differences in the Chinese carbon market by E-GARCH model, the method of comparison is based on the parameters of variance equation estimated by E-GARCH. Specifically, it is based on α and γ [40]. All the parameters are shown in Table 5.

**Table 5:** Parameters of variance equation.

	ω	α	γ	β
Coefficient	-1.6816	0.1652	0.0992	0.7788
P value	0.0000	0.0000	0.0000	0.0000

**Fig 5:**
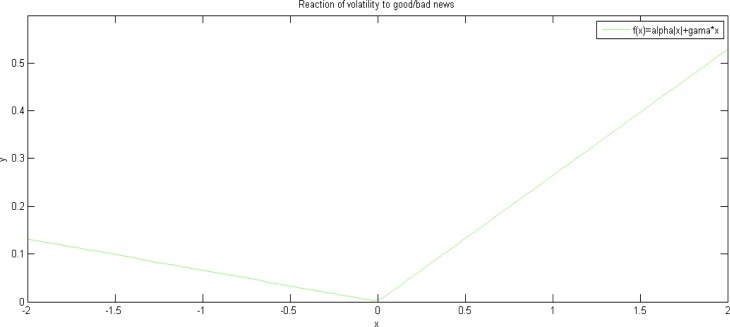
Reaction of volatility to good/bad news.

The graph for the function of y = f(x) = α|x| + γx can be used to confirm the difference [40], which is shown in Fig 5.

In Fig 5, x > 0 means good news, and x < 0 means bad news. As we can see from Fig 5, significantly asymmetry exists in the Chinese carbon market, as the slope is significantly different in the two parts of x > 0 and x < 0. Moreover, it can be seen, that the impact of bad news is smaller than that of good news, which is different from common stock markets.

## Discussion

As shown in Fig 3, the carbon market fluctuates severely at the beginning of carbon trading, but then becomes stable, despite several dramatic changes. The following reasons may account for the results. When the Chinese government begins to implement the carbon emission scheme in Shenzhen, the entities constrained by carbon trading need some time to become familiar with this new trading market [41–42]; meanwhile, some constrained companies may be unwilling to be incorporated with ETS, which can be accounted by the lack of responsibilities on carbon reductions and the consideration on production strategies [43]. Both the two aspects result to the extreme inactive participations of carbon trading, accounting for the violent fluctuation at the beginning of carbon trading. Awareness and perceptions of carbon trading increase with the development of carbon market. First, some companies may realize the benefits of carbon trading spontaneously through the taxes and subsidies brought by carbon trading. Second, companies face pressure from the government. According to Lin’s [23] research, the Chinese carbon market is still an authority-manipulated system, and policies are introduced to this emerging market continuously, these policies, not only improve the market transparency, but also increase the invisible pressures on enterprises to participate in carbon trading. At the same time, other pilots begin to trade. All these aspects lead the trading volume to increase nationally, resulting the volatility to decreases. If one company is allocated with some allowances, it will meet the ‘yearly compliance events’, which are implemented by the government during March to September. A large amount of trading volume, i.e., ‘Agreement Volume’ is concentrated either before or after the event [44]. Although the number of transaction is small, the transaction volume and the turnover are huge. The giant but concentrated deals compared with normal time cause huge fluctuations in market prices. The above aspects account for the peaks during the time period of ‘yearly compliance events’. The substantial decline indicates the achievements in the Chinese carbon market. The fluctuation during ‘yearly compliance events’ may cause the policy makers to pay more attention to means of transactions. Besides, such volatility is of great significance for entities and invertors constrained by carbon trading to avoid risk under the current carbon conditions.

According to Fig 4, Table 4, and the Hurst index, we confirm that long-term memory exists in the volatility series of the carbon market in China. This may be explained by the following reasons. Jones [45] and Akerlof [46] point out that, under a sluggish economic environment, which is represented by a negative average return ratio in the Chinese carbon market, it is difficult to revert to equilibrium when the system receives an external shock as the transaction is inactive, thus resulting in long-term memory. According to the Kyoto Protocol, Clean Development Mechanism (CDM) programme is the only way for developing countries to participate in international carbon reductions, which causes the domestic carbon pilots to be almost separated from the international carbon market, resulting in the difficulty of spillovers to the international carbon markets when the pilots encounter great shocks. Therefore, the fluctuation can only be digested internally, contributing to the long-term memory to a certain degree. As we have mentioned above, the Chinese carbon market is still an authority-manipulated system [23], and the government makes the carbon market to be ‘policy-driven’ by continuously introducing new roles to the market. These policies contain similar information, resulting a similar reaction. The above aspects may account for the long-term memory in volatility series in the Chinese carbon market. The long-term memory also reminds the government to pay more attention to market stability when it decides either to introduce new policies or alter existing policies.

As can be seen from Fig 5, significant asymmetry exists in the Chinese carbon market. Good news causes greater shocks to volatility compared with bad news. This may be explained by the following reason. At present, constrained entities, rather than individual investors, have achieved a dominant position in the Chinese carbon market. Based on their own requirements, polluters can make an application for allowances to the administrative department in charge. Entities tend to over-apply, thus making allowances being over-allocated throughout the nation but not in each single pilot, which means that there are certain pilots and entities have insufficient allowances [47]. When the government increases the punishment for over emission of carbon dioxide, it is a bad news for the market because such action increases illegal costs of entities. However, the potential increase on costs may be offset by the over-allocated carbon permits in certain pilots [22]. Thus, the government action will not change the volatility by too much for such markets with extra allowances. For other pilots with insufficient allowances, an increase in the punishment will rise their volatilities. On the other hand, when the government decreases such punishment, it tends to be a good news for the market because of lower illegal costs. For both kinds of markets with extra or insufficient allowances, lower costs tend to decrease their motivations to buy permits, which will accordingly result in higher volatilities. As a result, the market reacts to good news more than bad news.

## Conclusions

In this paper, we focus on the volatility of the Chinese carbon market. The novelty of this paper lies in the integration of the seven carbon emission pilots that China has established in her mainland. To the best of our knowledge, this is the first research into the characteristics of the overall volatility of the carbon market in China. We use the E-GARCH, the series correlation test, the ADF-KPSS and R/S analysis to assess volatility. The results are summarized as follows. First, at the beginning of carbon trading, volatility is great, but then it becomes stable, despite some dramatic changes. Second, long-term memory exists in volatility series of the carbon market in China. Third, asymmetry exists in the Chinese carbon market, and the volatility is strongly affected by good news rather than bad news. The findings provide recommendations for policy makers in China and other developing countries either for commencing carbon trading or improving the current market.

More basic work is needed to improve the market performances. First a better understanding of carbon trading is desperately needed for enterprises to alter the fact that the currently driving force for carbon trading is ‘yearly compliant events’ rather than either investment for profits or carbon reduction. Second, strengthening international cooperation to create favourable conditions for shocks to diffuse, as long-term memory exists in the volatility series. Moreover, the government should reduce the number of policies and regulations which contain similar meanings. Third, individual investors. As stated by Ibrahim & Kalaitzoglou [48], individual investors have certain effects on the carbon market, and the government should create satisfying conditions to appeal individual investors, this may do some changes for the existing asymmetry.
